# Triple Procedure for Dense Cataractous Neovascular Glaucoma Patients

**DOI:** 10.1155/2020/1251203

**Published:** 2020-06-21

**Authors:** Hossam M. Moharram, Shaaban Abd-Elhamid Mehany Elwan, Mahmoud M. Nassar, Mohamed F. Abdelkader

**Affiliations:** Ophthalmology Department, Faculty of Medicine, Minia University, Minia, Egypt

## Abstract

**Purpose:**

One of the most difficult refractory glaucomas is the neovascular type (NVG), and its association with dense cataract adds to this difficulty. This study aimed to provide results of the triple surgical procedure for such conditions.

**Methods:**

12 eyes of 12 patients with NVG and dense cataract were included in this case series study. The mean age of patients was 57.25 ± 5.9 years. The mean preoperative intraocular pressure (IOP) was 47.25 ± 4.04 mmHg with maximum antiglaucoma therapy. The mean best corrected distant visual acuity (BCDVA) in LogMAR was 2.13 ± 0.38. All patients received intravitreal injection of 1.25 mg (0.05 ml) bevacizumab followed by phacoemulsification, pars plana vitrectomy (PPV) including panretinal photocoagulation (PRP), and trabeculectomy with mitomycin C (MMC). Mean IOP and BCDVA changes were the main outcome results of this study.

**Results:**

The follow-up period was 2 years. The mean BCDVA was improved to 1.22 ± 0.35, 1.13 ± 0.34, 1.12 ± 0.37, 1.06 ± 0.38, and 1.01 ± 0.37 at 1, 3, 6, 12, and 24 months, respectively, after this procedure. This improvement was statistically significant when compared with preoperative BCDVA (*P* < 0.0001). The mean postoperative IOP was dropped to 20.08 ± 4.1, 17.08 ± 2.1, 17.17 ± 5, 15.75 ± 4.7, and 16.17 ± 6.1 mmHg, respectively. At the last follow-up, the mean IOP was statistically significantly lower than preoperative IOP (*P* < 0.0001) at the previously mentioned time points. The success rate was complete in 90.9% of eyes and qualified in 100% of eyes. Iris and angle neovascularization had regressed significantly in all patients, and no serious complications occurred during the follow-up period.

**Conclusions:**

This triple surgery can safely improve patients with NVG and dense cataract regarding BCDVA and IOP control. This trial is registered with NCT04143620.

## 1. Introduction

Ocular ischemia due to proliferative diabetic retinopathy (PDR), and central retinal vein occlusion (CRVO) is the main cause that contributes to the development of neovascular glaucoma (NVG) [1, 2]. Ischemic retina derived factors, such as the vascular endothelial growth factor (VEGF) affect the anterior segment and initiate neovascularization in the iris (NVI) and neovascularization in the angle (NVA) [3]. Aqueous outflow is obstructed when the neovascular fibrous tissue blocks the trabecular meshwork and leads to synechial angle closure; thus, NVG develops [4]. On the other hand, intraocular pressure (IOP) rise due to NVG lowers the ocular perfusion leading to further retinal ischemia, and this in turn induces more neovascularization. The management of NVG is very difficult because conventional treatments such as antiglaucoma drugs, trabeculectomy, cyclophotocoagulation, and cyclocryotherapy have poor success rates [5, 6]. Shunt procedures or glaucoma drainage implants (GDI) are considered the mainstay surgical treatment in this group of patients; however, different studies revealed variable success rates [7]. It is very important to promptly reduce the ischemic drive for the treatment of NVG. Panretinal photocoagulation (PRP) is mandatory and effective in resolving the ischemic drive and decreasing the production of vasoproliferative factors [8, 9]. This management is particularly difficult in the eyes with dense cataract. However, it is possible to overcome this difficulty by doing phacoemulsification and pars plana vitrectomy (PPV) with PRP. Therefore, a successful treatment plan must address all concurrent pathologies and ideally should include the following procedures: anti-VEGF, phacoemulsification, PRP, and antiglaucoma surgery. The introduction of pars plana vitrectomy (PPV) to the list of procedures has proven its effectiveness particularly if the media is not clear [10]. Moreover, phacoemulsification combined with PPV enables us to apply PRP from the posterior pole to the ora serrata peripherally. It is known that mitomycin C (MMC) increases the success rate of trabeculectomy in patients with NVG [11]. Therefore, in the current study, we performed intravitreal bevacizumab (IVB) injection, phacoemulsification, PPV, PRP, and trabeculectomy augmented with subconjunctival injection of MMC.

The aim of this study was to evaluate the safety and efficacy of this combined surgical procedures to alleviate retinal ischemia, reduce IOP, and improve visual acuity in patients with dense cataract and NVG.

## 2. Subjects and Methods

Twelve eyes of 12 patients (7 males and 5 females) with NVG associated with dense cataract enough to obscure fundus visualization were included in the study in the period from April 2016 to October 2019 at Ophthalmology Department, Faculty of Medicine, Minia University. Surgery for all eyes was performed in the first year of the study, and the follow-up was continued for two years. The age of patients ranged from 47 to 66 years (mean 57.25 ± 5.9 years). The underlying cause for NVG was PDR in 8 eyes (66.67%) and CRVO in 4 eyes (33.33%). Vitreous hemorrhage was present in half (50%) of the patients. The study was approved by the Local Ethical Review Committee and adhered to the tents of Declaration of Helsinki. All patients signed a written consent after discussion of the potential benefits and risks of this triple surgical procedures.

### 2.1. Inclusion Criteria

The study included patients with dense cataract and uncontrolled NVG with the maximum tolerated antiglaucoma medications.

### 2.2. Exclusion Criteria

The exclusion criteria included eyes with previous antiglaucoma surgery, silicone oil-filled eyes, previous buckle surgery or conjunctival scaring from any cause, eyes with clear crystalline lens or faint cataract, eyes with corneal opacity, and eyes with visual acuity less than the hand motion with a good perception of light.

### 2.3. Preoperative Examinations

Full ophthalmological examinations were performed including history taking, age, gender, laterality, etiology of NVG, and number of used antiglaucoma drugs. The ocular examination included estimation of best corrected distance visual acuity (BCDVA), IOP measurement with a Goldman applanation tonometer, slit-lamp examination of the anterior segment, and gonioscopy examination of angle of anterior chamber, biometry, and ultrasonography. The demographic data are registered in Table 1.

### 2.4. Surgical Procedure

All procedures were carried out under peribulbar anesthesia with mild systemic sedation. IVB injection of 1.25 mg (0.05 ml) was given 2–6 days before surgery using a 27-gauge needle at the inferotemporal quadrant at 3.5–4.0 mm posterior to the limbus. To lower IOP before surgery, preoperative intravenous mannitol was given to all cases in addition to the full antiglaucoma drugs including topical dorzolamide-timolol combination BID, brimonidine tartrate TID, and oral acetazolamide tablet (250 mg) TID. Subconjunctival injection of MMC in a dose of 0.04 mg/ml was carried out, and a period of 4 minutes was left before conjunctival opening.

Fornix-based conjunctival incision was performed, and a rectangular scleral flap of 3 × 4 mm was dissected. Phacoemulsification was performed through a separate temporal clear corneal incision with implantation of a one-piece hydrophobic IOL into the capsular bag, and the incision was closed with a 10/0 nylon suture. This was followed by a three-port 25-G PPV including core vitrectomy, injection of triamcinolone acetonide, induction of PVD, shaving of vitreous base, and dealing with any epiretinal membranes. PRP using diode endo-laser was performed up to the far periphery (2000–3000 shots, duration 200 ms; power 400 mw). Fluid-air exchange was then performed, and 20% SF6 was injected leaving 10 cc of gas to adjust pressure at the end of surgery. Then, the upper sclerotomies were sutured by Vicryl 7/0, and the infusion cannula was left in place connected to the syringe of 20% SF6. Then, Healon was injected into the anterior chamber to maintain the depth of anterior chamber, and trabeculectomy by Kelly punch and peripheral iridectomy were performed. Scleral flap was sutured by two 10/0 nylon sutures at the corners followed by watertight conjunctival wound closure. More SF6 was injected to adjust IOP, and the infusion cannula was removed, and its site was sutured with a Vicryl 7/0 suture. At the end of surgery, fluid was injected into the AC to test for filtration of bleb and to make sure that the conjunctiva was closed watertight. At the end of surgery, the subtenon injection of triamcinolone acetonide was given to all eyes.

### 2.5. Postoperative Management

All patients were given prednisolone acetate 1% (Pred Forte, Allergan Co.) eye drops QID and tapered through 8 weeks, cyclopentolate 0.5% eye drops TID, moxifloxacin 0.3 mg (Vigamox, Alcon Co.) eye drops QID for 2 weeks, and ointment of tobramycin and dexamethasone (Tobradex, Alcon Co.) at night for 4 weeks. Scheduled follow-up visits were the 1^st^ postoperative day, one week, two weeks, every month for three months, and then, every three months for 2 years.

Postoperatively, full ophthalmic examination was performed including BCDVA, IOP, gonioscopy, slit-lamp examination, and dilated fundus examination. Antiglaucoma medications were prescribed if IOP was more than 21 mmHg. Baseline results and that of 1, 3, 6, 12, and 24 months were included in the statistical analysis. The main outcome measures of this study were the mean BCDVA (LogMAR), the mean IOP, and the incidence of complications. Successful surgery was considered when IOP ≤21 mmHg was achieved without serious complications such as suprachoroidal hemorrhage, retinal detachment, endophthalmitis, phthisis bulbi, or persistent hypotony (IOP <5 mmHg). Complete success was considered when IOP of 8–21 mmHg was achieved without any antiglaucoma drugs and qualified success when this target IOP was achieved with and without the use of antiglaucoma drugs. Failure was defined as IOP >21 mmHg despite the use of maximum tolerated antiglaucoma medications, the occurrence of hypotony, or other serious ocular complications.

### 2.6. Statistical Analysis

Statistical analysis was performed with SPSS 19. Data were expressed as mean ± standard deviation (SD). Changes in the mean BCDVA and the mean IOP were compared for each follow-up visit with baseline using the paired *t*-test, and construction of graphs was performed by using Graph Pad Prism 5 program. A *P* value <0.05 was considered statistically significant.

## 3. Results

### 3.1. Best-Corrected Distant Visual Acuity

**Figure 1:**
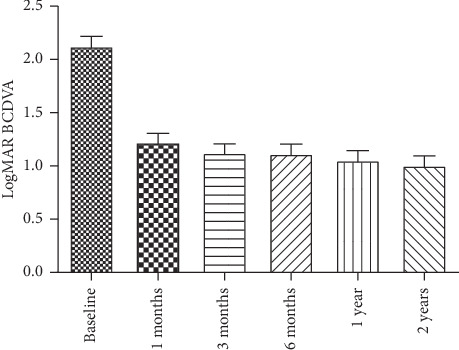
The mean BCDVA changes in LogMAR.

The mean LogMAR of BCDVA of the 12 eyes was 2.13 ± 0.38 at baseline and markedly improved at 1, 3, 6, 12, and 24 months postoperatively where the mean LogMAR of BCDVA was 1.22 ± 0.35, 1.13 ± 0.34, 1.12 ± 0.37, 1.06 ± 0.38, and 1.01 ± 0.37, respectively. Differences between preoperative and postoperative values throughout the follow-up visits were statistically significant (*P* < 0.0001). Vision was improved in 8 out of 12 eyes (66.67%), stable in 2 eyes (16.66%), and decreased in 2 eyes (one of them had optic nerve atrophy, and the other had sustained IOP elevation with refractory glaucoma that underwent the glaucoma drainage implant (GDI) surgery (Table 2 and Figure 1).

### 3.2. Intraocular Pressure Changes

**Figure 2:**
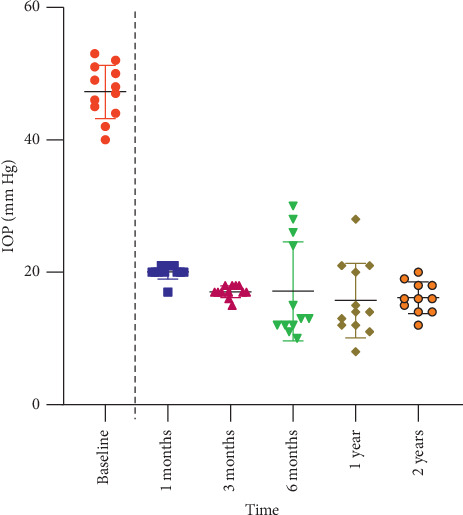
Intraocular pressure changes overtime.

Table 3 and Figure 2 demonstrate the patients' mean baseline IOP (47.25 ± 4.04 mmHg), which was decreased postoperatively to 20.08 ± 4.1, 17.08 ± 2.1, 17.17 ± 5, 15.75 ± 4.7, and 16.17 ± 6.1 mmHg at 1, 3, 6, 12, and 24 months, respectively. The mean postoperative IOP was significantly reduced when compared with baseline IOP at the previous follow-up visits (*P* < 0.0001).

### 3.3. Bleb Morphology

In the early postoperative period, all patients had diffuse filtering blebs, but in the 6th month, 2 eyes had shallow blebs with high IOP, which were treated medically. One eye had encapsulated bled, which was treated by bleb needle revision, and 1 eye had flat bleb, which was treated by GDI surgery.

### 3.4. Success Rate and Number of Antiglaucoma Drugs

Within the first 3 months postoperative follow-up, all patients achieved complete success and IOP between 8 and 21 mmHg without treatment. This, complete success, decreased on the 6^th^ month postoperative follow-up to 66.67%; 4 eyes (33.33%) had high IOP, 2 eyes were controlled by medical treatment, and the other 2 eyes (16.66%) had medically uncontrolled high IOP: one of them was treated by bleb needle revision and the other one underwent GDI surgery. The addition of antiglaucoma treatment resulted in 83.33% of patients achieving a qualified success (target IOP reached with antiglaucoma treatment). The success rate increased again and reached to 83.33% for complete success and 91.66% for qualified at a one-year follow-up visit, and it reached to 90.9% for complete success and 100% for qualified at 2 years follow-up visit. One eye lost the last follow-up visit (Table 4). The number of antiglaucoma drugs was significantly decreased from 3.66 ± 0.9 to 0.6 ± 0.2 at six months and to 0.1 ± 0.09 at last the follow-up (*P*=0.001 and 0.0001) (Table 5).

### 3.5. Gonioscopy and Neovascularization

Preoperative gonioscopy showed neovascularization of iris and angle with variable degrees of peripheral anterior synechiae (PAS), but none of our eyes had 360 degrees of PAS.

One week after the IVB injection, the neovascularization in the iris regressed in all patients. Gonioscopy performed one month postoperatively showed that the neovessels disappeared from the iris and angle in all cases, and the iridectomy and trabeculectomy sites could be seen in the upper quadrant. During the whole follow-up visits, no eyes had recurrent iris or angle neovascularization. Posterior segment neovascularization as documented by fluorescein angiography recurred in 3 eyes (25%) that needed augmentation PRP after 9–13 months postoperatively.

### 3.6. Intraoperative and Postoperative Complications

All procedures went without significant intraoperative complications. As shown in Table 6, in the early postoperative period, there was fibrinous iritis in 5 eyes (41.66%), which resolved by an increasing frequency of corticosteroid drops for the first postoperative week. Mild corneal edema was present in 6 eyes (50%) and resolved spontaneously in the first postoperative week. Mild hyphema was present in 4 eyes (33.33%) and resolved spontaneously in the first 3 postoperative days. Shallow anterior chamber was present in 1 eye (8.33%) with spontaneous improvement in the first postoperative week. One case (8.33%) had complete optic nerve atrophy. Posterior capsular opacification occurred in 9 (75%) of eyes, which was treated with YAG posterior capsulotomy. No cases had choroidal effusion, suprachoroidal hemorrhage, or retinal detachment. There were no hypotony, bleb-associated infection, or corneal decompensation. Also, endophthalmitis and phthisis bulbi were not found in our study.

## 4. Discussion

NVG is a serious complication of ocular ischemia, and it is very difficult to manage especially in the presence of dense cataract or vitreous hemorrhage obscuring visualization of the posterior segment. NVG eyes have a high level of VEGF in their ocular fluids, and its inhibition by intravitreal injection of anti-VEGF plays an important therapeutic role in treatment of NVG [12]. As previous studies documented the usefulness of IVB in regression of neovascularization for management of NVG [13–15], all patients in this study received IVB injection before surgery to suppress the neovascularization and increase the success rate of this triple procedure for NVG associated with dense cataract.

Our results showed that NVI and NVA regressed in all patients following IVB injection providing better conditions for surgery to improve its outcome. Studies reported that MMC decreased the activity of fibroblasts, lowered the postoperative fibrosis, and decreased the incidence of filtration failure [16–18]. Subconjunctival injection of MMC was used to augment the rate of success of trabeculectomy in our patients. Anti-VEGF agents produce temporary regression of neovascularization, which allowed us to further control retinal ischemia and neovascularization by PRP. However, all our patients had corneal edema from uncontrolled high IOP and dense cataract, 50% of them had vitreous hemorrhage, and 66.67% of eyes had PDR with epiretinal membranes making PRP impossible to do. Therefore, this triple procedure including phacoemulsification, PPV with PRP, and trabeculectomy with MMC allowed us to address all concurrent pathologies.

In this study, removal of cataract by phacoemulsification allowed accurate visualization of the posterior segment for complete PPV and application of adequate PRP. In our procedure, great care was taken during phacoemulsification to preserve the posterior capsule to prevent VEGF migration from posterior segment anteriorly. PPV was important not only to remove vitreous hemorrhage but also to eliminate VEGF and cytokines, deal with the epiretinal membranes in cases of proliferative diabetic retinopathy, and improve retinal circulation. PRP was important to lower retinal ischemia, prevent the formation of VEGF, and decrease the incidence of retinal detachment. In addition to its role in permanent lowering of IOP, trabeculectomy helped to overcome the transient early postoperative IOP elevation following cataract surgery and PRP, thus avoiding further optic nerve damage. Kinoshita et al. concluded that incomplete PRP to the peripheral retina and incomplete PPV can cause extensive fibrinous vitritis and worsening the NVI [19]. However, we did not face these complications in our study as we did complete vitrectomy as well as full PRP till the ora serrata. In our study, 20% SF6 gas was used for retinal tamponade to prevent postoperative hypotony and suprachoroidal hemorrhage. The gas was completely absorbed after mean time of 17.41 ± 4 days, and these complications were not encountered in our study.

The mean postoperative BCDVA was significantly improved to 1.01 ± 0.37 LogMAR at 2 years, and the difference from baseline was statistically significant at every follow-up visit. The BCDVA LogMAR improved in 8 eyes (66.67%) and remained stable in 2 eyes (16.66%). Our results were similar to the results of Li et al. [20] who reported that at 12 months after PPV, pars plana lensectomy (PPL), PRP, and trabeculectomy, the mean LogMAR of BCDVA was 1.26 ± 0.29, and this difference was statistically significant when compared with the mean LogMAR of preoperative BCDVA of 2.62 ± 0.43 (*P*=0.002), and the LogMAR of BCDVA improved in 22 eyes (84.62%) and remained stable in 4 eyes (15.38%). However, they used pars plana lensectomy (PPL) for different degree of cataract density with IOL implantation in the sulcus, preoperative intravitreal injection of ranibizumab, and a shorter period of follow-up (1 year), but in our study, we used temporal corneal incision phacoemulsification for all our cases, which had dense cataract with IOL implantation in the capsular bag, preoperative IVB, and a longer follow-up period of 2 years.

Kolomeyer et al. [21] performed combined PPV and Baerveldt tube insertion for NVG patients. Forty-five (51%) 20-gauge, 12 (13%) 23-gauge, and 32 (36%) 25-gauge pars plana vitrectomies were performed with fifty-two eyes (58%) preoperatively received intraocular injections. Their LogMAR visual acuities at 18, 24, 36, and 48 months follow-up were significantly better than preoperative vision (*P* < 0.05), and preoperative versus final IOP and number of glaucoma medications were significantly decreased (*P* < 0.05). Fourteen eyes (16%) had visual acuity of no light perception. They reported that the frequency of postoperative complications were significantly (*P* < 0.05) higher in 20-gauge versus 23/25-gauge pars plana vitrectomy eyes. 4 eyes (4.5%) developed retinal detachment, and 3 (3.4%) had high IOP due to tube occlusion. Three (3.4%) developed endophthalmitis, and 2 (2.2%) progressed to being pre/phthisical. In our study, all eyes received preoperative IVB; we used 25-gauge in all eyes, and no retinal detachment or endophthalmitis occurred. Their study was retrospective and had a large sample size (89 eyes) with variable NVG severity at baseline and a longer follow-up period, and surgeries were performed by multiple retinal and glaucoma specialists. Also, another difference between our technique and theirs was that we did complete PRP (2000–3000 shots). In fact, proper PRP alleviates retinal ischemia, and hence neovascularization, and preserves vision in NVG patients.

Demircan [22] performed combined treatment of IVB, PRP, and diode laser cyclodestruction (DLCD) in 27 eyes with NVG. Their baseline LogMAR of visual acuity was 2.62 ± 0.76 and improved to 2.44 ± 0.87, and their mean IOP was 44.1 ± 11 and decreased to 18.1 ± 3.4 mmHg. However, 6 eyes out of 27 (22.2%) underwent a second DLCD due to high IOP, and one eye was complicated by hypotony. They had a shorter follow-up period of 6.7 ± 0.7 months with a different procedure than ours.

In our study, the combined procedure of IVB, phacoemulsification, PPV, complete PRP, and trabeculectomy with MMC have effectively prevented patients' visual loss and improved their vision. The mean IOP in our study was reduced at the last follow-up visit to 16.17 ± 6.1 mmHg, which was significantly lower than the mean preoperative IOP of 47.25 ± 4.04 mmHg. Our target IOP was achieved for complete success in 83.33% and 90.9% and for qualified success in 91.66% and 100% at 1 and 2 years, respectively. These results were better than that reported by Li et al. study [20], in which their mean IOP decreased significantly from 46.38 ± 5.75 to 16.68 ± 2.96 mmHg. Their complete success was 65.38% and qualified one was 84.62% at the last follow-up visit; however, their follow-up period was only 12 months.

Kinoshita [19] studied the surgical results of combined PPV, PPL, PRP, and silicon oil tamponade for NVG. Their success rates for IOP ≤21 mmHg and sustained light perception were 92.3% at 3 months and 69.2% at 1 year. Our procedure differed from that of Kinoshita et al., in which we did trabeculectomy augmented with MMC, and we used SF6 gas instead of silicon oil tamponade. Trabeculectomy safeguarded against the early transient postoperative IOP elevation, which may be induced by cataract surgery and PRP. Also, silicon oil tamponade may elevate the IOP, which may result in further damage to the optic nerve, and hence visual loss.

Our results were in disagreement with the results of Artini et al. [9] who studied 18 eyes of NVG who underwent IVB injection and PRP, and the mean IOP was reduced from 39 ± 10.2 to 24.4 ± 8.0 mmHg (*P*=0.001) in one week and elevated again to 30.4 ± 6.7 mmHg. All eyes required additional glaucoma intervention (implants for 14 eyes and cyclocryotheraphy for 4 eyes). In our study, we addressed all the concurrent pathologies in one procedure.

## 5. Conclusions

In conclusion, although NVG with dense cataract is very hard to manage, our technique can be beneficial as it addresses multiple pathologies simultaneously increasing the chances to control IOP and preserve the remaining vision or even improve it. Our findings concluded that IVB, phacoemulsification, PPV, complete PRP, and trabeculectomy with MMC can control IOP and improve BCDVA without serious ocular complications for such patients. The weak point in our study is the limited number of patients; therefore, further studies with a large number of patients are still required to assess long-term safety.

## Figures and Tables

**Table 1:** Patients' demographic data.

Parameters	Discreption
Eyes (*n*)	12
Age (year) mean ± SD	57.25 ± 5.9
Range	(47–66)
Sex (M/F)	7/5
R/L eyes	8/4
Preoperative IOP (mm Hg)	
Mean ± SD	47.25 ± 4.04
Range	(40–53)
BCDVA (LogMAR) Underlying disease	2.13 ± 0.38
DM (*n*, %)	8 (66.67)
CRVO (*n*, %)	4 (33.33)
Axial length	22.09 ± 0.78
Mild corneal edema (*n*, %)	12 (100)
NVI and NVA	12 (100)
Dense cataract	12(100)
Ocular ultrasonography	
Vitreous hemorrhage	6 (50)
Coarse epiretinal membrane causing retinal traction	8 (66.67)

**Table 2:** The mean BCDVA changes in LogMAR.

	Baseline	1 month	3 months	6 months	1 year	2 years
Visual acuity	2.13 ± 0.38	1.22 ± 0.35	1.13 ± 0.34	1.12 ± 0.37	1.06 ± 0.38	1.01 ± 0.37
Difference		0.91	1	1.01	1.07	1.12
*P* value		<0.0001	<0.0001	<0.0001	<0.0001	<0.0001

**Table 3:** IOP (mmHg) changes overtime.

	Baseline	1 month	3 months	6 months	1 year	2 years
IOP	47.25 ± 4.04	20.08 ± 4.1	17.08 ± 2.1	17.17 ± 5	15.75 ± 4.7	16.17 ± 6.1
Difference		27.17	30.17	30.08	31.5	31.08
*P* value		<0.0001	<0.0001	<0.0001	<0.0001	<0.0001

**Table 4:** Success and failure rates over time.

	No. of eyes	Lost eyes	Complete success	Qualified success	Failure
One month	12	0	12 (100%)	12 (100%)	0
Three months	12	0	12 (100%)	12 (100%)	0
Six months	12	0	8 (66.67%)	10 (83.33%)	2
One year	12	0	10 (83.33%)	11 (91.66%)	1
Two years	11	1	10 (90.9%)	11 (100%)	0

**Table 5:** The mean number of antiglaucoma medications.

	Mean no.	*P* value
Preoperative	3.66 ± 0.9	
One month	—	—
Three months	—	—
Six month	0.6 ± 0.2	0.001
One year	0.3 ± 0.25	0.002
Two years	0.1 ± 0.09	0.0001

**Table 6:** Postoperative complications.

	Frequency	Percentage (%)
Iritis	5	41.66
Mild corneal edema	6	50
Mild hyphema	4	33.33
Hypotony	0	00
Shallow anterior chamber	1	8.33
Anterior segment neovascularization	0	00
Posterior capsular opacification	9	75
Posterior segment neovascularization	3	25
Choroidal effusion	0	00
Supra choroidal hemorrhage	0	00
Optic nerve atrophy	1	8.33
Retinal detachment	0	00

## Data Availability

The datasets used and/or analyzed during the current study are available from the corresponding author upon reasonable request.

## Abbreviations

NVG: Neovascular glaucoma
IOP: Intraocular pressure
DR: Diabetic retinopathy
CRVO: Central retinal vein occlusion
CRAO: Central retinal artery occlusion
OIS: Ocular ischemic syndrome
NVI: Neovascularization of the iris
NVA: Neovascularization of the angle
BCDVA: Best corrected distant visual acuity
IVB: Intravitreal bevacizumab
PPV: Pars plana vitrectomy
PRP: Pan retinal photocoagulation
LP: Light perception
GDI: Glaucoma drainage implant
VEGF: Vascular endothelial growth factor
MMC: Mitomycin C
SO: Silicon oil
IV: Intravenous
SF6: Sulphur hexafluoride gas
QID: Four times/day
TID: Tree times/day
BID: Two times/day
A/C: Anterior chamber.
